# ATXN2-Mediated PI3K/AKT Activation Confers Gastric Cancer Chemoresistance and Attenuates CD8^+^ T Cell Cytotoxicity

**DOI:** 10.1155/2022/6863240

**Published:** 2022-09-28

**Authors:** Qi Wang, Tianyu Cao, Xiaohui Zhang, Juan Hui, Chen Wang, Wenyao Zhang, Pei Wang, Yun Zhou, Shuang Han

**Affiliations:** ^1^Honghui Hospital, Xi'an Jiaotong University, Xi'an, Shaanxi 710049, China; ^2^The 928 Hospital of PLA Joint Logistics Support Forces, Haikou, Hainan 570206, China; ^3^State Key Laboratory of Cancer Biology, National Clinical Research Center for Digestive Diseases and Xijing Hospital of Digestive Diseases, Xijing Hospital, Fourth Military Medical University, Xi'an, Shannxi 710038, China; ^4^Department of Gastroenterology, Tangdu Hospital, Fourth Military Medical University, 710038 Xi'an, China; ^5^Department of Gastroenterology, Ningxia Medical University, Yinchuan, Ningxia 750004, China

## Abstract

As one of the primary therapeutic choices, chemotherapy is widely adopted for progressive gastric cancer (GC), but the development of chemoresistance has limited chemotherapy efficacy and partly contributes to poor prognosis. Immunotherapy is increasingly being applied in the clinical treatment of GC and is also benefitting patients. To ascertain whether ATXN2 affects chemotherapy efficacy in GC cells and its role in GC immune escape, we performed high-throughput sequencing to clarify genes differentially expressed between 5-FU-resistant and 5-FU-sensitive GC cells and then conducted qRT–PCR to assess ATXN2 expression in GC tissues. Furthermore, the influence of ATXN2 on resistance was studied in vitro and *in vivo*, ATXN2 and other protein expression levels were detected using Western blotting and immunohistochemistry (IHC), and the direct association of SP1 and ATXN2 was confirmed through luciferase reporter gene analysis. We found elevated ATXN2 in GC tumors and a negative correlation between ATXN2 levels and the prognosis of GC. Furthermore, by activating the PI3K/AKT pathway, ATXN2 was found to promote chemoresistance in GC, facilitating BCL2L1 expression. In GC cells, ATXN2 further stimulated PD-L1 expression and provided better immunotherapy efficacy. Finally, we demonstrated that SP1 transcriptionally regulated the expression of ATXN2 and prompted GC chemoresistance and immune escape. In conclusion, our study reveals the important roles of the SP1/ATXN2/PI3K-AKT/BCL2L1 signalling pathway in GC chemoresistance and of the SP1/ATXN2/PI3K-AKT/PD-L1 signalling pathway in GC immunotherapy. Our findings provide new theories and experimental references for overcoming chemotherapy resistance in GC and enhancing the efficacy of immunotherapy for GC.

## 1. Introduction

Gastric cancer (GC), the cells of which compose one of the most common malignant tumors, ranks fifth in incidence and fourth in mortality worldwide [1]. The current primary therapy for GC consists of surgical resection, chemotherapy, radiotherapy, and targeted therapy. Since GC often develops asymptomatically, most diagnoses are advanced [2]. Chemotherapy is considered a routine treatment for advanced GC [3]. Unfortunately, its clinical efficacy is limited due to severe side effects and chemoresistance [4]. Glimelius et al. confirmed a median overall survival of 8 months with conventional chemotherapy for advanced-stage GC [5].

The development of tumors involves a complex series of processes, including interactions between cancer cells and the host immune system. Immunotherapy, defined as targeting the immune system to treat disease, has emerged as a promising therapy with fewer adverse reactions and drug resistance than current chemotherapies [6]. The immune checkpoint (IC), which is represented by a group of inhibitory pathways of immune cells, including cytotoxic T lymphocyte-associated antigen-4 (CTLA-4) and programmed death-1 (PD-1), can control the persistence of the immune response while maintaining the body's self-tolerance [7]. ICs are highly correlated with the initiation of immune cell signalling pathways, through which tumor cells can evade immune surveillance, thus forming a microenvironment conducive to tumor development [8]. Evidence suggests that tumor cells evade antigen-specific T cell immune responses by upregulating IC ligands, such as PD-L1 and PD-L2, on their surfaces [9]. Immune checkpoint inhibitors (ICIs), which block the IC pathway to reinvigorate the antitumor immune response, have become an indispensable part of cancer immunotherapy [7, 10, 11]. PD-1/PD-L1 inhibitors have been shown to rehabilitate T cell activity, strengthen the body's immune response, and effectively recognize and kill tumor cells, thereby achieving long-term remission of tumor patients [12, 13]. As a classic PD-1 inhibitor, nivolumab has been applied clinically in an increasing number of diseases since it was first approved for treating unresectable or metastatic melanoma in December 2014 [14]. Encouragingly, several clinical studies have shown that nivolumab has achieved lasting efficacy and significant clinical benefits in patients with advanced GC [15–17].

ATXN2, an evolutionarily conserved RNA-binding protein in eukaryotes, is physiologically located in the endoplasmic reticulum and Golgi apparatus, regulates mRNA translation and protein synthesis, and participates in the stress response [18, 19]. However, when cells are subjected to pathological conditions such as damage and energy loss, ATXN2 transcription is enhanced, and ATXN2 mRNAs are translocated to a site referred to as the stress particle, where mRNAs are protected from translation and degradation [20, 21]. Given that ATXN2 can regulate nerve excitability and even circadian rhythm, modulate the endocytosis of trophic receptors and growth pathways, and exert strong effects on mitochondrial precursor proteins and metabolic enzymes, most of the related reports on ATXN2 have been focused on neuro-related and metabolism-related diseases [22–27]. Overexpression of ATXN2 correlated with the proliferation and metastasis of pancreatic cancer [28]. However, to date, the roles and potential mechanisms of ATXN2 in the progression of GC and chemoresistance remain poorly understood.

In this work, we show that ATXN2 is strongly expressed in GC and induces chemoresistance by activating the PI3K/AKT signalling pathway. In addition, ATXN2 can facilitate PD-L1 expression, and silencing ATXN2 results in improved effectiveness of immunotherapy. Our study reveals the dual role of the ATXN2-PI3K/AKT pathway in chemoresistance and immunotherapy, which will contribute to a better understanding of the interaction between chemoresistance and immunotherapy. Our study suggests that targeting SP1 and ATXN2 can alleviate chemoresistance and promote immune drug efficacy. Our study provides new ideas and strategies for clinical chemoresistance and immunotherapy resistance.

## 2. Results

### 2.1. ATXN2 Is Associated with Chemoresistance and Prognosis of GC

**Figure 1:**
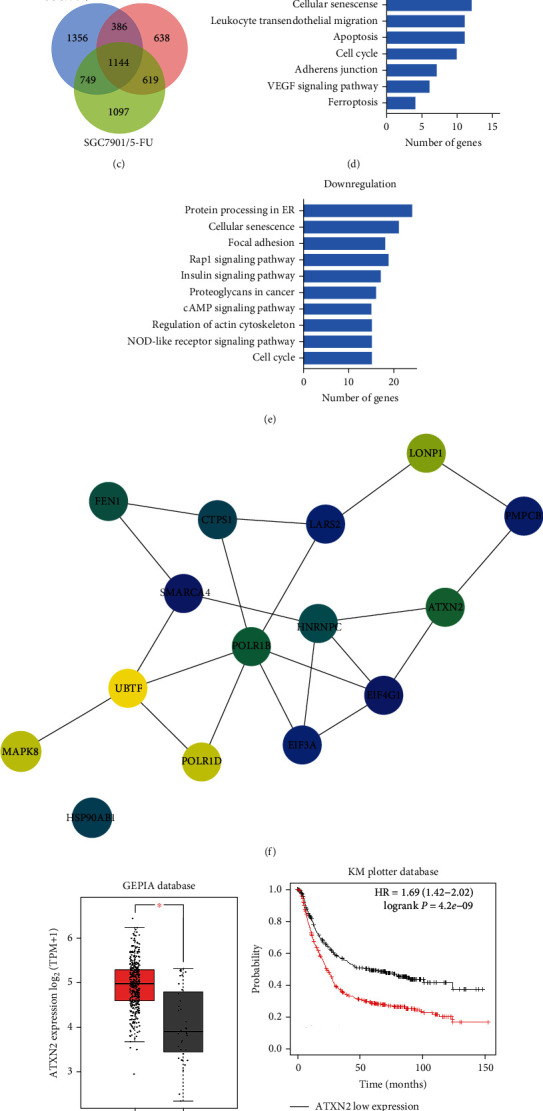
Identification of differentially expressed genes between chemoresistant and chemosensitive cell lines. (a) Heatmap of the altered genes in SGC7901/ADR, SGC7901/VCR, and SGC7901/5-FU cells versus SGC7901 cells. (b, c) The differentially upregulated (b) and downregulated (c) genes in chemoresistant cells. (e, f) The KEGG pathways for the downregulated (d) and upregulated (e) in chemoresistant cells. (f) The top 15 hub genes according to betweenness centrality, eccentricity, and stress algorithms. (g) ATXN2 expression in the GEPIA database and UALCAN database. (h) The association of ATXN2 expression with GC prognosis. (i) The association of ATXN2 expression with GC clinical stage. ^∗^*P* < 0.05 and ^∗∗^*P* < 0.01.

We screened differentially expressed genes from the high-throughput sequencing data of SGC7901 cells and three lines of chemoresistant GC cells (Figure 1(a)). In total, 889 and 1114 genes were upregulated and downregulated, respectively, in the chemoresistant GC cells (Figures 1(b) and 1(c)). KEGG pathway enrichment analysis revealed significant changes in signalling pathways such as “cell cycle” and “apoptosis” (Figures 1(d) and 1(e)). Furthermore, protein–protein interaction (PPI) network analysis based on the sequencing data consisted of 277 nodes and 603 edges (Figure S1). Fifteen of the central nodes were screened from 277 nodes according to betweenness centrality, eccentricity, and stress algorithms (Figure 1(f)). We then examined the expression and prognosis of the 15 hub genes in GC with the GEPIA database and Kaplan–Meier Plotter database and discovered upregulated ATXN2 expression in GC with the highest hazard ratio (HR = 1.69) (Figures 1(g) and 1(h), Figure S2A, B). Furthermore, ATXN2 expression was closely associated with GC clinical stage (Figure 1(i)). Therefore, we selected ATXN2 for further study.

### 2.2. High ATXN2 Expression Promotes Chemoresistance in GC

**Figure 2:**
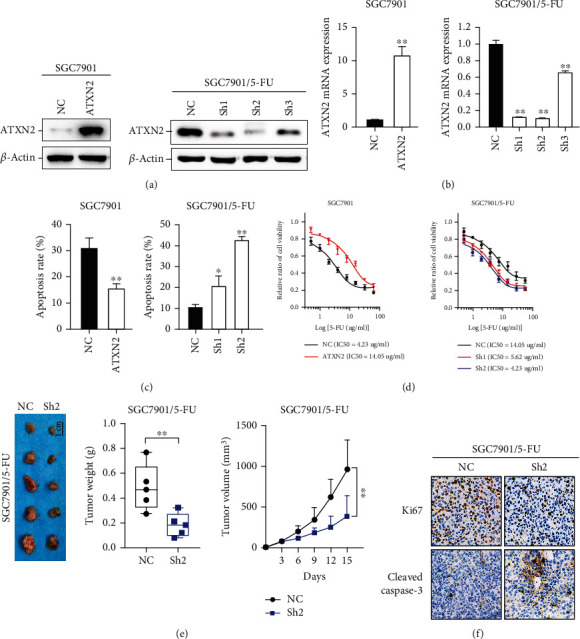
High ATXN2 expression promoted chemoresistance in GC. (a, b) ATXN2 upregulation in SGC7901 cells and ATXN2 downregulation in SGC7901/5-FU cells were verified at the protein (a) and mRNA (b) levels. (c) SGC7901 cells overexpressing ATXN2 and SGC7901/5-FU cells with downregulated ATXN2 were treated with 5-FU (10 *μ*g/mL). The apoptosis rate of the two cell lines was detected. (d) The IC_50_ of SGC7901 cells overexpressing ATXN2 and SGC7901/5-FU cells with downregulated ATXN2. (e) SGC7901/5-FU cells with downregulated ATXN2 or vector control were transplanted into nude mice, and the weight and volume of tumors were measured. (f) IHC analysis of Ki67 and cleaved caspase-3 expression. ^∗^*P* < 0.05 and ^∗∗^*P* < 0.01.

The effects of ATXN2 on GC chemoresistance were examined using gain/loss-of-function models in vitro (Figures 2(a) and 2(b)). The results of the cell apoptosis assay indicated that upregulation of ATXN2 reduced apoptosis induced by 5-FU in SGC7901 cells, while downregulation of ATXN2 promoted apoptosis (Figure 2(c)). Moreover, when ATXN2 was upregulated, the IC_50_ (for 5-FU) of SGC7901 cells increased; when ATXN2 was downregulated, the IC_50_ (for 5-FU) of drug-resistant GC cells decreased (Figure 2(d)). The results of animal models showed that knocking down ATXN2 decreased the tumor weight and volume of nude mice treated with 5-FU (Figure 2(e)). Immunohistochemical staining of nude mouse tumor tissues showed that knockdown of ATXN2 inhibited ki67 expression and promoted cleaved caspase-3 expression (Figure 2(f)). In summary, these results suggest that ATXN2 exhibits remarkable antiapoptotic and chemoresistance abilities.

### 2.3. ATXN2 Promotes Drug Resistance through Activation of the PI3K/AKT/BCL2L1 Pathway

**Figure 3:**
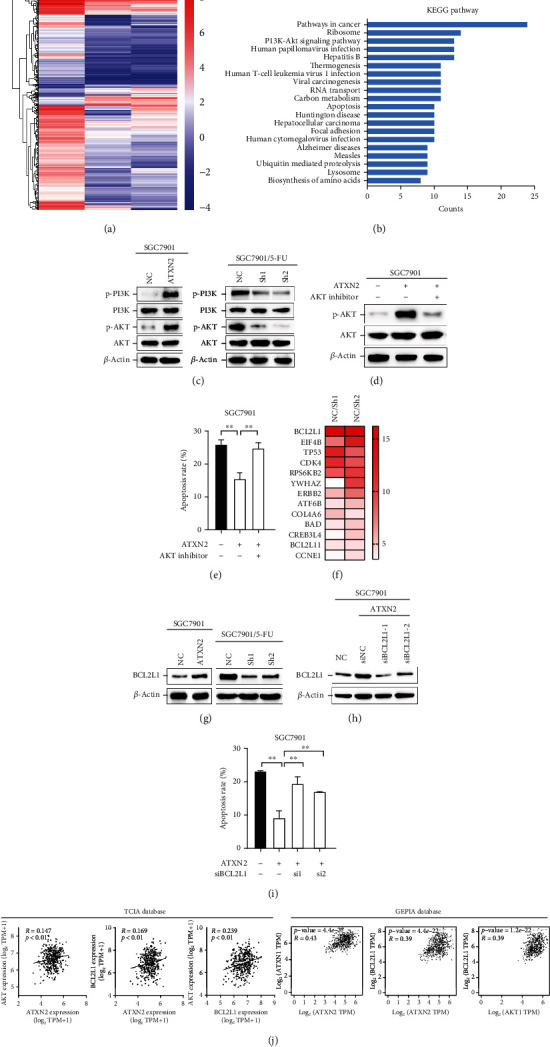
ATXN2 activated the PI3K/AKT pathway. (a) Heatmap of altered genes when ATXN2 was downregulated in SGC7901/5-FU cells. (b) KEGG pathway analysis of changed pathways when ATXN2 was downregulated in SGC7901/5-FU cells. (c) Phosphorylation levels of PI3K and AKT were measured by Western blot. (d) Protein levels of p-AKT and AKT in SGC7901 cells overexpressing ATXN2 or treated with an AKT inhibitor. (e) Apoptosis rates of SGC7901 cells overexpressing ATXN2 or treated with an AKT inhibitor were detected after treatment with 5-FU (10 *μ*g/mL). (f) Downregulated genes enriched in the PI3K/AKT pathway. (g) Protein level of BCL2L1 in SGC7901 cells and SGC7901/5-FU cells. (h) Protein level of BCL2L1 in SGC7901 cells overexpressing ATXN2 and/or silencing BCL2L1. (i) Apoptosis rates of SGC7901 cells overexpressing ATXN2 or (and) silencing BCL2L1 were detected after treatment with 5-FU (10 *μ*g/mL). (j) The correlation between ATXN2, AKT, and BCL2L1 expression in the TCIA and GEPIA databases. ^∗∗^*P* < 0.01.

High-throughput sequencing was performed on SGC7901/5-FU and downregulated ATXN2 cells, and differentially expressed genes were identified (Figure 3(a)). ATXN2-silenced SGC7901/5-FU cells showed pathway expression profiles that differed from those of the control cells, in which the PI3K-AKT pathway was among the strongly downregulated pathways (Figure 3(b)). Next, we verified that the phosphorylation of PI3K and AKT significantly increased when ATXN2 was upregulated and decreased when ATXN2 was downregulated (Figure 3(c)). We then found that an AKT inhibitor (AKT-IN-1) was capable of counteracting the reduced cell apoptosis induced by 5-FU resulting from upregulation of ATXN2 (Figures 3(d) and 3(e)). Furthermore, we analysed the genes enriched in the PI3K/AKT signalling pathway and identified BCL2L1 as the gene with the greatest variation (Figure 3(f)). The protein encoded by BCL2L1 belongs to the BCL-2 family, of which the family members form dimers and are involved in various cellular activities as antiapoptotic regulators. ATXN2 regulated the expression of BCL2L1 (Figure 3(g)), and downregulation of BCL2L1 blocked the increase in cell apoptosis caused by ATXN elevation (Figures 3(h) and 3(i)). Finally, analysis of the contents of the TCIA and GEPIA databases showed that ATXN2 expression positively correlated with BCL2L1 and AKT in GC (Figure 3(j)).

### 2.4. ATXN2 Increases PD-L1 Expression in GC Cells

**Figure 4:**
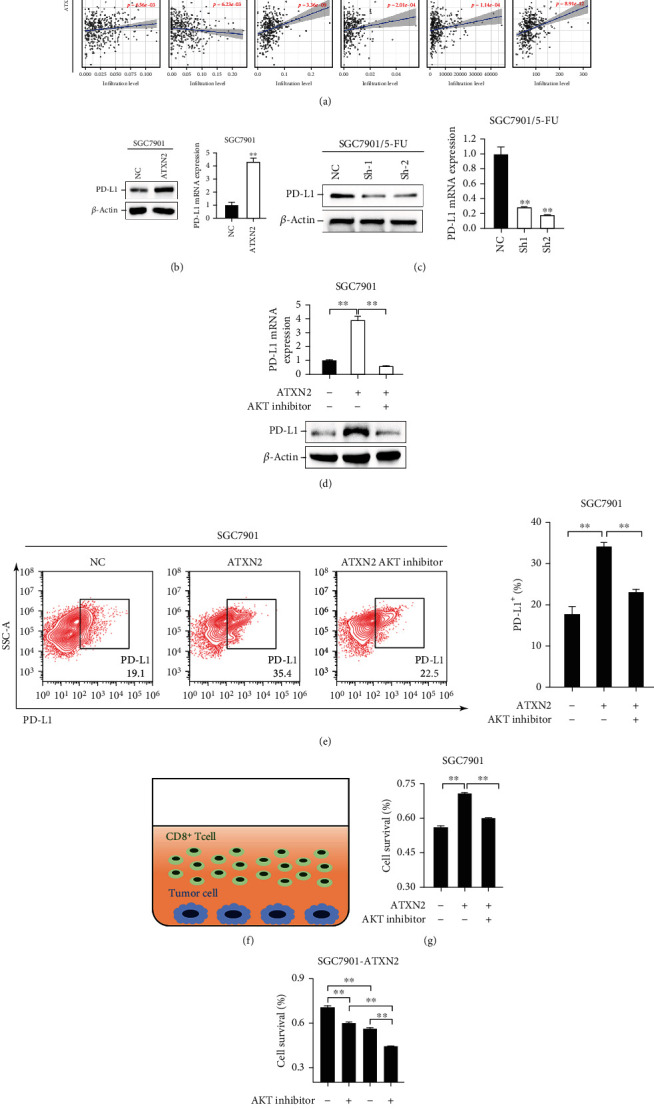
ATXN2 increased the expression of PD-L1. (a) Correlation analysis of ATXN2 expression and immune cell infiltration. (b, c) Protein and mRNA levels of PD-L1 in SGC7901 cells (b) and SGC7901/5-FU cells (c). (d) Protein and mRNA levels of PD-L1 in SGC7901 cells overexpressing ATXN2 or treated with an AKT inhibitor. (e) Flow cytometric analysis of PD-L1 expression in SGC7901 cells overexpressing ATXN2 or treated with an AKT inhibitor. (f) Coculture of CD8^+^ T cells and tumor cells. (g) ATXN2 was overexpressed in SGC7901 cells treated with an AKT inhibitor and cocultured with CD8^+^ T cells. The survival rates of SGC7901 and SGC7901-ATXN2 cells were measured. (h) SGC7901-overexpressing ATXN2 cells were treated with an AKT inhibitor or nivolumab and cocultured with CD8^+^ T cells. The survival rate of SGC7901-ATXN2 cells was measured. ^∗∗^*P* < 0.01.

By analysing the TIMER database, we found that ATXN2 was closely associated with the infiltration of immune cells in GC (Figure 4(a)). Since PD-L1 is a well-known IC and PD-L1 is regulated by PI3K/AKT [29], we explored the interaction between ATXN2 and PD-L1. ATXN2 overexpression promoted PD-L1 expression, and ATXN2 knockdown inhibited PD-L1 expression (Figures 4(b) and 4(c)). Application of an AKT inhibitor largely offsets the increase in PD-L1 expression caused by ATXN2 overexpression (Figures 4(d) and 4(e)). We then cocultured CD8^+^ T cells with GC cells and added an AKT inhibitor and nivolumab as treatments (Figure 4(f)). Upregulation of ATXN2 reduced CD8^+^ T cell killing capacity, which was reversed by supplementation with an AKT inhibitor (Figure 4(g)). Moreover, in CD8^+^ T cells, both AKT inhibitors and nivolumab enhanced the killing capacity, and the drug combination enabled the strongest killing capacity (Figure 4(h)). In summary, the above findings indicated that ATXN2 leads to PD-L1 elevation by activating the PI3K/AKT pathway, subsequently modulating immunotherapy efficacy.

### 2.5. SP1 Transcriptionally Activates ATXN2 in GC

**Figure 5:**
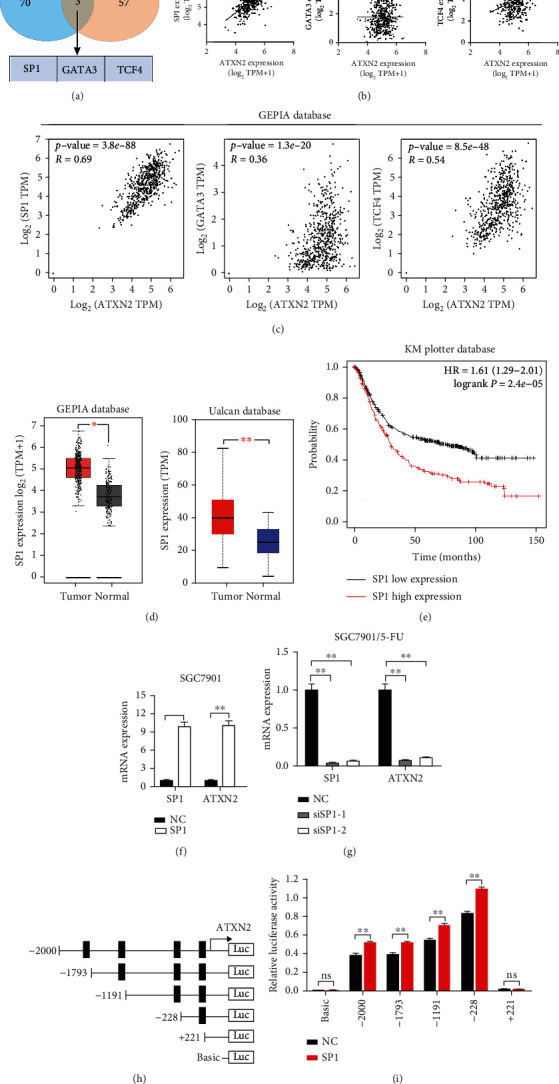
SP1 regulated the expression of ATXN2. (a) Transcription factors were identified using the JASPAR and PROMO databases. (b, c) The correlation between ATXN2 and SP1 and GATA3 and TCF4 in the TCIA database (b) and GEPIA database (c). (d) The expression of SP1 in the GEPIA database. (e) The correlation between SP1 and GC prognosis. (f, g) SP1 and ATXN2 expression in SGC7901 cells (f) and SGC7901/5-FU cells (g). (h, i) Dual luciferase reporter assay analysis of SP1 binding to the ATXN2 promoter. ^∗∗^*P* < 0.01.

**Figure 6:**
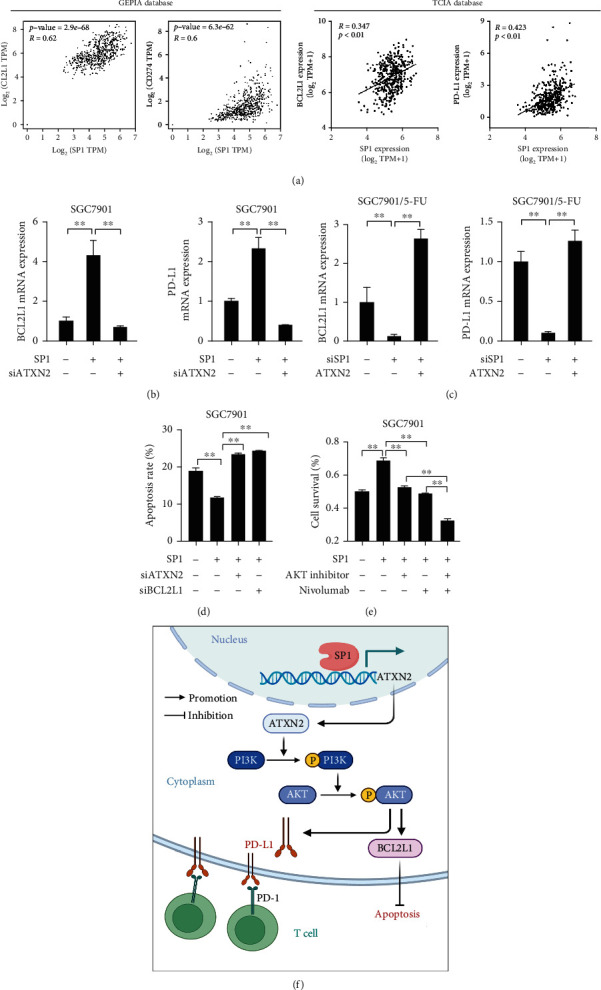
SP1 affected chemoresistance and immunotherapy. (a) Correlation analysis of SP1, BCL2L1, and PD-L1 in the GEPIA and TCIA databases. (b, c) The expression of BLC2L1 and PD-L1 in SGC7901 cells (b) and SGC7901/5-FU cells (c). (d) The apoptosis rate of SGC7901 cells with SP1 overexpression, ATXN2 silencing, and BCL2L1 silencing. (e) SGC7901 cells overexpressing SP1 were treated with an AKT inhibitor or nivolumab and cocultured with CD8^+^ T cells. The survival rate of SGC7901 cells was measured. (f) Schematic of the model for the SP1/ATXN2/PI3K-Akt/BCL2L1 and SP1/ATXN2/PI3K-Akt/PD-L1 pathways. The schematic was created with http://biorender.com. ^∗∗^*P* < 0.01.

To explore the cause of elevated ATXN2 expression, we analysed the contents of the JASPAR database and PROMO database and identified three transcription factors, namely, SP1, GATA3, and TCF4 (Figure 5(a)). We then conducted a correlation analysis and found that the strongest correlation occurred between SP1 and ATXN2 in both the TCIA database (Figure 5(b)) and the GEPIA database (Figure 5(c)). The elevated SP1 of GC was shown in the GEPIA database and was related to a poor GC prognosis (Figures 5(d) and 5(e)). The results from SGC7901 cells showed that upregulating SP1 promoted ATXN2 expression. Furthermore, SP1 silencing inhibited ATXN2 expression in SGC7901/5FU cells (Figures 5(f) and 5(g)). In addition, a dual-luciferase reporter assay demonstrated the binding of SP1 to the ATXN2 promoter region (Figures 5(h) and 5(i)). Furthermore, we analysed the correlation between SP1, BCL2L1, and PD-L1 in the GEPIA and TCIA databases and found that SP1 was positively related to BCL2L1 and PD-L1 (Figure 6(a)). We cotransfected SP1-expressing plasmids and ATXN2-targeting siRNA into SGC7901 cells and discovered that SP1 overexpression increased BLC2L1 and PD-L1 expression, which could be blocked by ATXN2 silencing (Figure 6(b)). In addition, we cotransfected SP1-targeting siRNA and ATXN2-expressing plasmids into SGC7901/5-FU cells and found that SP1 silencing decreased BLC2L1 and PD-L1 expression, which could be offset by ATXN2 overexpression (Figure 6(c)). The apoptosis assay showed that SP1 upregulation reduced the cell apoptosis rate in response to 5-FU, which was reversed by ATXN2 silencing or BCL2L1 silencing (Figure 6(d)). Finally, we transfected SP1-expressing plasmids and added an AKT inhibitor or nivolumab into SGC7901 cells, which were then cocultured with CD8^+^ T cells. The cell survival assay indicated that overexpression of SP1 decreased the response towards CD8^+^ T cell killing, whereas an AKT inhibitor and nivolumab increased the response (Figure 6(e)). The combination of an AKT inhibitor and nivolumab showed the strongest response to CD8^+^ T cell killing. In conclusion, these results suggest that SP1 transcriptionally regulates ATXN2 expression, which activates the PI3K-AKT/BCL2L1 and PI3K-AKT/PD-L1 pathways to influence chemoresistance and immunotherapy (Figure 6(f)).

## 3. Materials and Methods

### 3.1. Cell Culture

The GC cell line SGC7901 was purchased from the American Type Culture Collection (ATCC). SGC7901/5-FU, SGC7901/ADR, and SGC7901/VCR cells were obtained through stepwise screening with VCR, ADR, and 5-FU in our laboratory. All cells were cultured in Dulbecco's modified Eagle's medium (Gibco, USA) containing 10% foetal bovine serum (Gibco, USA).

### 3.2. Reagents

5-Fluorouracil was obtained from MicroCode Engineering (MCE, USA). The AKT inhibitor (AKT-IN-1) and nivolumab were purchased from MCE (USA). All reagents were added according to the manufacturer's recommendation.

### 3.3. RNA Extraction and qPCR

RNA extraction was performed in accordance with the recommendation from Qiagen (Germany). The PCR primers were as follows: ATXN2, forward 5′-TTGATGCCGCACATGAGAAAA-3′ and backward 5′-CGCCATTCACTTTAGCACTGAT-3′; SP1, forward 5′-AGTTCCAGACCGTTGATGGG-3′ and backward 5′-GTTTGCACCTGGTATGATCTGT-3′. Reverse transcription and qPCR were conducted using kits from TaKaRa (Japan). Gene expression was measured with a LightCycler 480 system (Roche, Switzerland). Each experiment was repeated independently at least three times.

### 3.4. Protein Extraction and Western Blots

Protein extraction from cells was performed using RIPA lysis buffer. The protein concentration was determined, and protein denaturation was conducted by boiling for 10 minutes. Thirty micrograms of protein was used for electrophoresis. After blocking with 10% skim milk, the protein membranes were immersed in primary antibody (4°C, overnight) followed by incubation with secondary antibody for 1 hour. The protein membranes were subjected to chemiluminescence. The antibodies used were as follows: anti-ATXN2 from Santa Cruz (USA) and anti-*β*-actin, anti-PI3K, anti-p-PI3K, anti-AKT, anti-p-AKT, anti-BCL2L1, and anti-PD-L1 from CST (USA).

### 3.5. Immunohistochemistry (IHC)

Immunohistochemical staining was performed using anti-Ki67 and anti-Cleaved caspase 3(CST, USA) antibodies according to the manufacturer's recommendation. The ratio (positive cell number to total cell number) was calculated to indicate the expression score.

### 3.6. Dual-Luciferase Reporter Assays

Bioinformatics methods were used to predict and analyse the possible transcription factor-binding sites of ATXN2. Then, the DNA fragment containing the ATXN2 promoter in the human genome was amplified according to the primer sequence and plugged into the reporter vector. The reporter gene plasmid and SP1-expression plasmid were cotransfected into SGC7901 cells. Finally, the luciferase activity was determined, and the relative fluorescence intensity was calculated.

### 3.7. IC_50_ Assay

After the plating and attachment of 3000 cells, PBS or 5-FU was added to every well of 96-well plates. CCK-8 (Abcam, USA) was diluted and added to 96-well plates for 2 h of incubation. The absorbance value was measured with a Multimode Reader (Bio-Rad, USA). The IC_50_ value was then calculated according to the number of viable cells in the PBS or 5-FU groups.

### 3.8. Apoptosis Detection

Cells were trypsinized, aspirated, centrifuged, and then evenly mixed in PBS. Cells (100 *μ*L) were transferred into a flow tube with Annexin V and 7-AAD reagents, and incubation was maintained for 30 minutes under dark conditions. The cell suspension was examined via flow cytometry after being washed with PBS.

### 3.9. *In Vivo* Tumorigenicity

Subcutaneous transplantation of target cells (1 × 10^6^ cells) into the backs of Balb/c nude mice was performed to observe *in vivo* tumorigenicity. After the tumor volume reached 100 mm^3^, PBS or 5-FU was injected into the abdominal cavity of the mice. After the mice were euthanized, xenograft tumors were isolated for further analysis.

### 3.10. CD8^+^ T Cell Activation and Coculture

CD8^+^ T cells from STEMCELL (USA) were activated using a specialized T cell activator (Catalogue # 10971) as recommended, collected, cocultured with GC cells for 24 h, and washed off. The survival rate of GC cells was measured with CCK-8.

### 3.11. Bioinformatics Analysis

Protein–protein interaction (PPI) network analysis was used to analyse the interaction between proteins. The GEPIA database, KM plotter database, UALCAN database, and TCIA database were used to analyse the expression and clinical prognosis of genes. The TIMER database was used to analyse the relationship between ATXN2 and immune cell infiltration. The JASPAR database and PROMO database were used to predict the transcription factors of ATXN2.

### 3.12. Statistical Analyses

The SPSS software (version 23.0) was employed for data analysis. Means ± standard deviations (SDs) were used to represent continuous data. Student's unpaired *t*-test or one-way ANOVA was performed to statistically analyse the difference between two groups or one-way multigroups. *P* < 0.05 was regarded as statistically significant.

## 4. Discussion

With approximately 480,000 new cases in 2020, GC has become the third most prevalent malignancy in China [1]. As chemotherapy remains the primary treatment choice for progressive GC, chemoresistance has a more prominent impact on GC treatment compared with other cancers and is the most problematic issue in our clinical work, which greatly affects patient survival [3]. The Ataxin-2 protein encoded by ATXN2 is a protein involved in RNA metabolism and metabolic homeostasis [30]. In our study, we revealed that ATXN2 has proliferative and antiapoptotic effects and that a high ATXN2 level was related to an adverse prognosis. Elevated ATXN2 expression facilitated 5-FU resistance in GC cells, which was decreased by downregulating ATXN2. Additionally, ATXN2 enhanced BCL2L1 expression (an antiapoptotic factor) by activating PI3K/AKT signalling, ultimately resulting in 5-FU resistance.

Immune cells within the tumor microenvironment (TME) are involved in tumorigenesis development [31]. Immune cells, especially infiltrating T cells, can recognize tumor antigens and participate in killing tumor cells [32]. However, clinically detected cancers often evade the antitumor immune response of immune cells. The ability of immune evasion is emerging as a new hallmark of cancer, unexpectedly providing an opportunity for a new strategy in cancer therapy, namely, the use of immune cells against cancer cells. Recently, IC modulators have shown unexpected antitumor effects in a variety of cancers, opening a new era in cancer therapy. PD-L1 is critical in physiological immune homeostasis and tumor immune escape [33]. The PI3K/AKT signalling pathway regulates PD-L1 expression in tumor cells [29, 34, 35]. Our results confirmed a close connection between ATXN2 and immune cell infiltration in GC and proved that elevated ATXN2 promoted PD-L1 expression. Furthermore, our results revealed that ATXN2 promotes PD-L1 expression by activating the PI3K/AKT pathway and that ATXN2 knockdown promoted the efficacy of immunotherapy.

The abundance of SP1, an important transcription factor, is typically increased in most tumors, and SP1 participates in tumor cell proliferation, differentiation, DNA damage response, apoptosis, senescence, and angiogenesis [36, 37]. Our work confirmed the binding between SP1 and the ATXN2 promoter region and the promotion of ATXN2 mRNA and protein expression by SP1. We also found that SP1 transcriptionally activated ATXN2, allowing this protein to participate in chemoresistance and escape from immune surveillance in GC. In summary, our study revealed that the SP1/ATXN2/PI3K-AKT/BCL2L1 pathway promotes GC chemoresistance and that the SP1/ATXN2/PI3K-AKT/PD-L1 pathway promotes GC immune escape. The correlation between tumor chemoresistance and immunity is not very clear. Our study linked chemoresistance to tumor immunotherapy and found that the key hub is the PI3K-Akt pathway. Inhibition of the PI3K-Akt pathway can significantly reduce chemoresistance and enhance the efficacy of immunotherapy. Our study will provide a reference for inhibitors of the PI3K-AKT pathway to address chemoresistance and improve the efficacy of immunotherapy. Our findings provide a potential therapeutic approach to address GC chemoresistance, as well as a new theoretical and experimental basis for immunotherapy of GC.

## Data Availability

The datasets generated during and/or analysed during the current study are available from the corresponding author on reasonable request.

## Supplementary Materials

Supplementary 1 Supplementary Figure 1: PPI network analysis of genes altered in GC chemoresistant cells according to STRING.

Supplementary 2 Supplementary Figure 2: the expression of 14 hub genes in GEPIA and the association with prognosis in KM plotter databases. (A) The expression of 14 hub genes in the GEPIA database. (B) The association of 14 hub genes with prognosis in KM plotter databases.
