# Supplementary figures and images for: ATXN2-Mediated PI3K/AKT Activation Confers Gastric Cancer Chemoresistance and Attenuates CD8+ T Cell Cytotoxicity
Source: J Immunol Res. 2022 Sep 28;2022:6863240. doi: 10.1155/2022/6863240 (PMC9535133; doi:10.1155/2022/6863240)

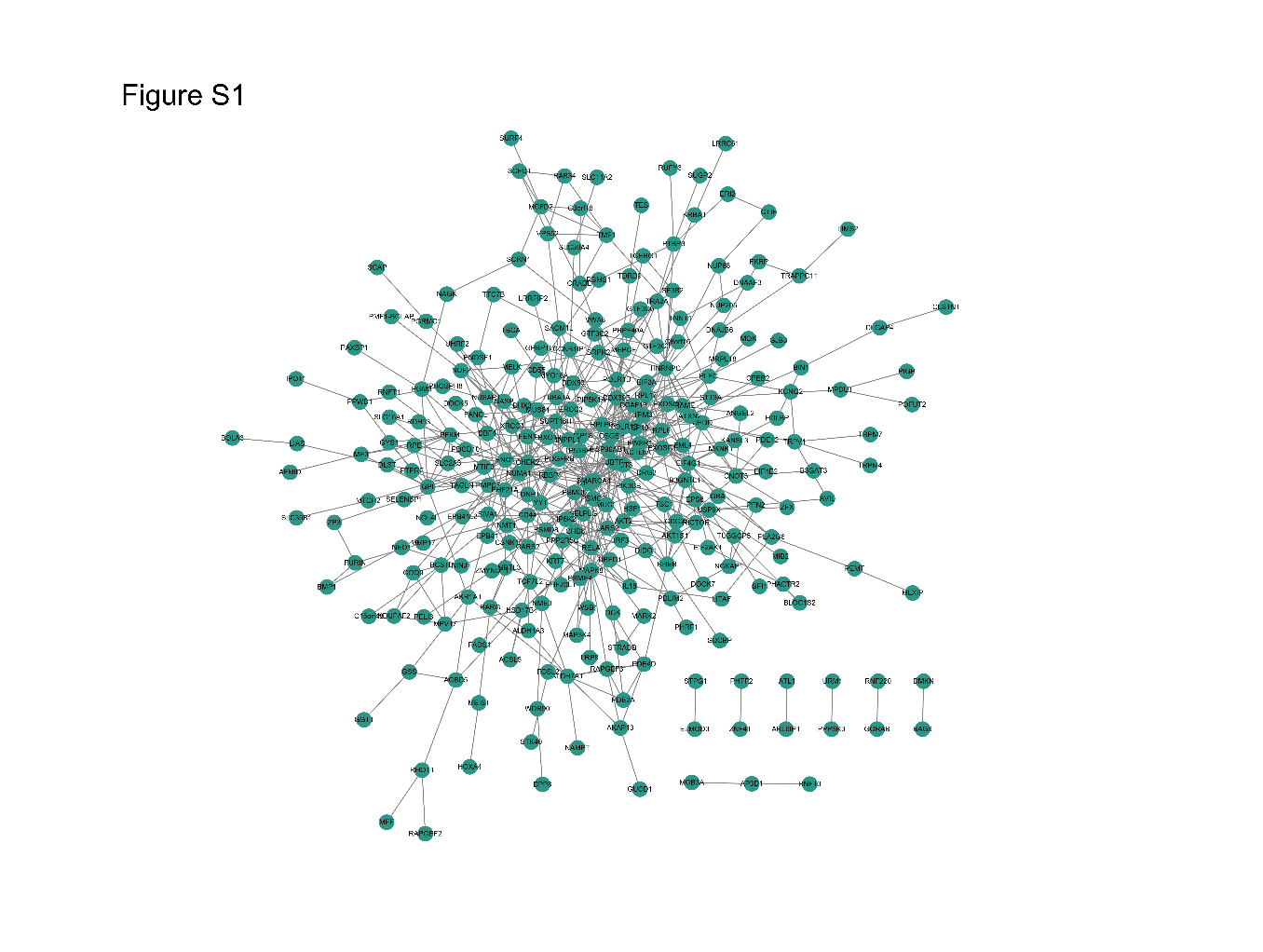

Supplement: Supplementary 1 — Supplementary Figure 1: PPI network analysis of genes altered in GC chemoresistant cells according to STRING. [file 6863240.f1.docx]

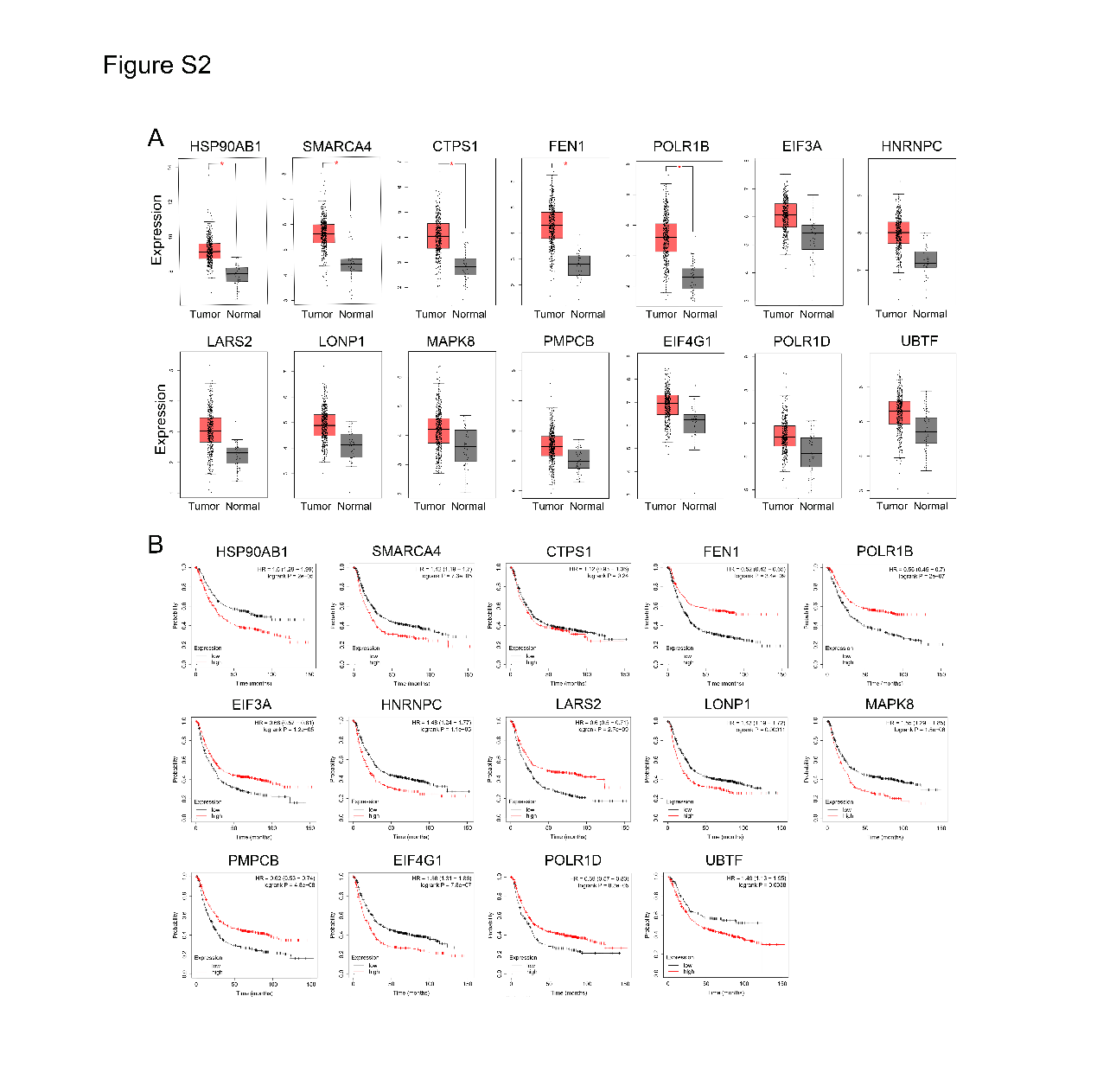

Supplement: Supplementary 2 — Supplementary Figure 2: the expression of 14 hub genes in GEPIA and the association with prognosis in KM plotter databases. (A) The expression of 14 hub genes in the GEPIA database. (B) The association of 14 hub genes with prognosis in KM plotter databases. [file 6863240.f2.docx]
